# Ellagic acid mitigates rotavirus-induced intestinal injury via bidirectional “immune-microbiota” regulatory effect

**DOI:** 10.3389/fcimb.2025.1686918

**Published:** 2025-12-29

**Authors:** Jiangang Zheng, Zhigang Cao, Wafa Yousaf, Abdul Haseeb, Ziyang Wang, Hejie Wang

**Affiliations:** 1 School of Public Health, Changzhi Medical College, Changzhi, Shanxi, China; 2 Shanxi Higher Education Institutions of Science and Technology Innovation Plan Platform, Laboratory of Environmental Factors and Population Health, Changzhi, China; 3 Key Laboratory of Environmental Pathogenic Mechanisms and Prevention of Chronic Diseases, Changzhi Medical College, Changzhi, China; 4 College of Veterinary Medicine, Shanxi Agricultural University, Shanxi, Jinzhong, China; 5 Institute of Traditional Chinese Medicine (TCM), Xinjiang Medical University, Urumqi, Xinjiang, China

**Keywords:** ellagic acid, rotavirus, BALB/c suckling mouse, TLR4, *Lactobacillus johnsonii*

## Abstract

**Introduction:**

Rotavirus (RV) is a major cause of childhood gastroenteritis, leading to intestinal damage, inflammation, and gut microbiota dysbiosis. This study investigated whether ellagic acid (EA), a natural polyphenol, can alleviate RV-induced intestinal injury by modulating both host immunity and the gut microbiota.

**Methods:**

In this study, RV was used to infect BALB/c suckling mouse models to explore whether ellagic acid could alleviate intestinal damage following rotavirus infection through bidirectional regulation of "immunity and microbiota". The viral load of RV, the expression levels of IL-1β, IL-6, and TNF-α mRNA were detected by qPCR. The pathological changes in the jejunal tissue were observed by hematoxylin and eosin (H&E) staining. The expression of JAM1, ZO-1, and Claudin-4 proteins in jejunal tissue were detected by immunohistochemistry (IHC). The expressions of TLR4, MYD88, IκBα, and P-P65 proteins in jejunal tissues were detected by WB. 16S rDNA gene sequencing was employed to detect the structural changes of the microbiota in feces, and qPCR was used to detect the colonization of Lactobacillus johnsonii, Lactobacillus reuteri, and Lactobacillus gasseri in jejunal tissues.

**Results:**

The qPCR results revealed that ellagic acid could significantly (P < 0.001) reduce the viral load as well as the mRNA expression levels of IL-1β, IL-6, and TNF-α in RV-infected BALB/c suckling mice. The results of H&E staining demonstrated that ellagic acid could alleviate villus rupture and vacuolation lesions induced by RV and significantly (P < 0.05) alleviate intestinal villus shortening and crypt deepening caused by RV. The IHC results showed that ellagic acid could significantly increase the expression of tight junction proteins JAM1, ZO-1, and Claudin-4 in RV-infected BALB/c neonatal mice. The WB results showed that ellagic acid significantly (P< 0.001) inhibited the expression of the TLR4/NF-κB signaling pathway. The results of 16S rDNA gene sequencing showed that ellagic acid could lead to a significant (P < 0.05) increase in the abundance of intestinal Lactobacillus bacteria (*Lactobacillus johnsonii*, Lactobacillus reuteri, *Lactobacillus gasseri*, etc.) in RV-infected BALB/c suckling mice. Ellagic acid can also significantly promote the colonization of Lactobacillus johnsonii, Lactobacillus reuteri, and Lactobacillus gasseriin the jejunum.

**Discussion:**

Ellagic acid can alleviate intestinal damage following rotavirus infection through bidirectional regulation of "immunity and microbiota", providing a theoretical foundation and innovative concepts for the research and development of EA as an anti-RV drug.

## 1 Introduction

Rotavirus (RV) is a non-enveloped virus belonging to the genus Rotavirus of the *Reovirus family* (Matthijnssens et al., 2022). Rotavirus infection is the leading cause of rotavirus gastroenteritis in children under 5 years old globally, resulting in approximately 120,000 pediatric fatalities annually and presenting a significant challenge to global public health. Rotavirus infection is usually self-limiting. As long as fluid is replenished in time, it often does not pose a hazard to life (Farthing, 2001). However, the intestinal epithelial damage caused by viruses can subsequently lead to inflammatory responses and intestinal flora disorders, resulting in persistent intestinal damage (Guerrero and Acosta, 2016; Zhao et al., 2021).

At present, there are four commercialized RV vaccines certified by the WHO (Rotarix, Rotateq, Rotavac, and Rotasiil), but rotavirus vaccines have not been included in the immunization programs of China and most other developing countries (Okafor and Ekwunife, 2021). Despite the extensive use of vaccines diminishing the prevalence of rotavirus, no specific medication exists for the treatment of rotavirus gastroenteritis. Consequently, the screening and development of pharmaceuticals are critically important (Gower et al., 2020). Traditional Chinese medicine often uses single or compound formulations containing pomegranate peel to treat diarrhea, which is recorded in ancient medical books such as “*Pu Ji Fang*” and “*Sheng Ji Zong Lu*”. Consequently, ellagic acid, a distinct constituent of pomegranate peel, is a possible anti-RV pharmacological agent. Research indicates that ellagic acid can alleviate diarrhea, suggesting significant promise as an anti-rotavirus agent and in mitigating associated intestinal damage (Chen et al., 2020a).

Rotavirus directly infects and destroys the mature intestinal epithelial cells at the apex of the small intestinal villi, leading to villi atrophy and causing osmotic diarrhea (Amimo et al., 2021). The rotavirus NSP4 protein activates chloride ion secretion through calcium signaling, causing secretory diarrhea (Chang-Graham et al., 2020). This can trigger a local inflammatory cascade reaction in the intestine, promoting the release of pro-inflammatory cytokines such as IL-6 and TNF-α, and further damaging the intestinal mucosal barrier (Jiang et al., 2003). In addition, after viral infection, probiotics (such as bifidobacteria) will decrease while potential pathogenic bacteria (such as Escherichia coli) will overgrow, establishing a vicious cycle (Gozalbo-Rovira et al., 2021; Bao et al., 2023; Yan et al., 2025).

In the initial stage of this study, TLR4 was screened out as a potential target of ellagic acid against RV by using network pharmacology and computational biology, and the specific binding between the two was verified by SPR (Zheng et al., 2024). Therefore, based on the verification of ellagic acid’s anti-RV activity, this study focuses on TLR4 as the key point to investigate the mechanism of ellagic acid’s anti-RV effect. TLR4 is a core pattern recognition receptor in the innate immune system, playing a crucial role in immune defense and inflammation regulation, and is also pivotal in maintaining intestinal homeostasis (Kumar et al., 2025). Inhibiting the TLR4/NF-κB signaling pathway can alleviate diarrhea caused by RV infection, indicating the feasibility of targeting TLR4 to inhibit intestinal damage induced by RV infection (Chen et al., 2020b). Ellagic acid possesses anti-inflammatory and antioxidant properties. Consequently, ellagic acid may inhibit intestinal inflammation and alleviate intestinal damage caused by RV infection by targeting TLR4 (Sharifi-Rad et al., 2022).

The duration of RV infection is relatively short; however, the secondary intestinal flora disorder (an increase in Gram-negative bacteria and a decrease in probiotics) can prolong the time frame of the disease and aggravate symptoms (Xiong et al., 2021). TLR4 plays a crucial role in intestinal immunity and mediates the recognition of LPS. Therefore, after rotavirus infection, the proliferation of secondary Gram-negative bacteria will activate TLR4-related pathways, exacerbating intestinal inflammation and damage (Lu et al., 2008). Ellagic acid is also widely utilized in the regulation of intestinal health, including regulating the intestinal flora, suppressing the proliferation of Gram-negative bacteria, promoting the growth of probiotics, facilitating the production of short-chain fatty acids (propionic acid and isobutyric acid), and enhancement of the intestinal barrier (Duan et al., 2022; Norouzalinia et al., 2025; Yang et al., 2025). Consequently, ellagic acid may alleviate the damage caused by RV infection not only by regulating the immune system but also by regulating the intestinal flora. Based on the RV-infected Balb/c mouse model, this study utilized technologies such as 16-s rRNA sequencing of intestinal microbiota to investigate how ellagic acid mitigates intestinal damage induced by RV infection through the bidirectional regulation of immunity and microbiota, establishing a theoretical basis for subsequent reduction of diarrhea duration and risk of complications by ellagic acid.

## 2 Materials and methods

### 2.1 Compounds, virus, and antibodies

Ellagic acid (Cat: HY-B0183) and Ribavirin (Cat: HY-B0434) were purchased from MCE (USA), with 99.75% and 99.96% purity, respectively.

Rhesus rotavirus (MMU 18006, 10^6.25^ TCID_50_/0.1mL) was purchased from ATCC (Cat: VR-1739).

Anti-Actin Rabbit pAb (Cat: GB15003), Anti-TBP Rabbit pAb (Cat: GB113810-100), Anti-TLR4 Rabbit pAb (Cat: GB11519-100), Anti-IκBα Rabbit pAb (Cat: GB111509-100), Anti-MYD88 Rabbit pAb (Cat: GB111554-100), and Anti-P-P65 Rabbit pAb (Cat: GB113882-100) were purchased from Servicebio (China). JAM-A/CD321/F11R Rabbit pAb (Cat: A1241) was purchased from ABclonal (China). Claudin 4-specific Polyclonal antibody (Cat: 16195-1-AP) and ZO-1 Polyclonal antibody (Cat: 21773-1-AP) were purchased from PTGLAB (USA).

### 2.2 Animal modeling and treatment

Four-day-old Balb/C suckling mice (weight 2.5 g) were purchased from SPF Biotechnology Co., Ltd (Beijing, China). The number of mice in each group was equal. The animals were raised in a controlled environment with a 12-hour light/dark cycle, with free access to food and water. All animal experiments were approved by the Ethics Committee of Changzhi Medical College (DW2025072, March 21, 2025) and were conducted under its guidance and regulations. After 3 days acclimatization period, the mice were randomly allocated into six groups: Control group; Model group (10^6^ TCID_50_/20 g); the high (100 mg/kg), medium (50 mg/kg), and low (25 mg/kg) dose groups of ellagic acid, and the positive drug ribavirin group (40 mg/kg), with 8 mice in each group, half male and half female. Twelve hours after RV infection (10^6^ TCID_50_/20 g), mice were treated with ellagic acid and ribavirin for 3 days. The 0.9% saline was used to dissolve the doses of ellagic acid and ribavirin. The control group was given an oral gavage of 0.9% saline equal to that of the other groups. Subsequent to the dissection, fixation, and cryopreservation of the jejunum tissue and the internal feces were collected, following euthanasia via CO_2_. The parameters of CO_2_ euthanasia are as follows: the chamber volume was 50×37×28 cm, the flow rate was 30% chamber volume/min, the CO_2_ concentration gradually increased to 90% of the chamber volume, and the treatment time was 5 minutes.

### 2.3 RNA extraction and quantitative real−time PCR

Total RNA was extracted from the jejunum tissue (25 mg), according to the Trizol protocol (Invitrogen, Carlsbad, CA, USA). The RNA concentration and purity were evaluated by using the Eppendorf BioPhotometer D30 (Eppendorf, USA). The complementary DNA was synthesized with PrimeScript^®^ RT Master Mix kit with gDNA Eraser (TaKaRa, Dalian, China) according to the manufacturer’s protocol. RT-qPCR was performed by using a 7500 Real Time PCR System (ABI, USA). Relative RT-qPCR was applied to detect the mRNA expression levels of *IL-1β*, *IL-6*, and *TNF-α* using the 2×SYBR Green qPCR Master Mix (Low ROX, Biotool, USA).

Relative expression levels were determined with the 2^-ΔΔCt^ method. Absolute RT-qPCR was applied to determine the RV VP6 gene; a standard curve was generated using serially diluted PCR amplification product. The primer sequence for the RV VP6 gene is as follows: F 5’-AGCGGTAGCGGCGTTATTT-3’, R 5’-ACTGGTCCAACTGGTAT CGC-3’; IL-1β F 5’-GTTGACGGACCCCAAAAGAT-3’ R 5’-TGATACTGCCTGCCTGAAGC-3’; IL-6 F 5’-GAGAC TTCCATCCAGTTGCCT-3’ R 5’-TGGGAGTGGTATCCTCTGTGA-3’; TNF-α F 5’-GCACAGAAAGCATGATCCGC -3’ R 5’-CCTGCCACAAGCAGGAATGA-3’; And β-actin F 5’-GTGCTATGTTGCTCTAGACTTC-3’ R 5’-ATGCCA CAGGATTCCATACC-3’; Lactobacillus johnsonii F 5’-CTAATGAGACTGCCGGTGACAAA-3’ R 5’-TCGCAGGTT CGCTTCTCGT-3’; Limosilactobacillus reuteri F 5’-GGCGGTTGCTTAGGTCTGA-3’ R 5’-TTCGCTACCCATGCT TTCG-3’; Lactobacillus gasseri 5’-ACGAAGCTGGAATCGCTAGTAATC-3’ R 5’-TTCACCCTAATCATCTGTCCT ACC-3’; GAPDH 5’-CCTCGTCCCGTAGACAAAATG-3’ R 5’-TGAGGTCAATGAAGGGGTCGT-3’.

### 2.4 Pathological and immunohistochemical analyses

Tissue samples of the mouse jejunum were fixed with 4% paraformaldehyde solution (Solarbio, China). The fixed tissue was dehydrated by a fully automatic dehydrator (JT-12S, Wuhan Junjie Electronics Co., LTD., China), later trimmed, embedded, and sliced into paraffin sections.

The hematoxylin and eosin (H&E) protocol (Solarbio, China) was performed to visualize pathological changes in jejunum tissue samples. Image acquisition was performed using the Pannoramic 250 digital slice scanner (3DHISTECH, Hungary). The intestinal tissue was selected for imaging using the CaseViewer2.4 scanning and browsing software. The Image-Pro Plus 6.0 analysis software was used uniformly with micrometers as the standard unit. The heights of 5 intact villi and the depths of 5 crypts in each section were measured, respectively, and the average values were calculated.

The expression of JAM1, ZO-1, and Claudin-4 in the jejunum was detected by immunohistochemistry (IHC). Paraffin sections were dewaxed with water and then treated with antigen repair, endogenous peroxidase blocking, and bovine serum blocking. JAM1, ZO-1, and Claudin4 primary antibodies (1:100, 1:1000, and 1:200) were applied, and the sections were placed in a wet box at 4°C and incubated overnight. Later, secondary antibody (HRP-labeled goat anti-rabbit, 1:100) was applied. DAB drops were added to the tissue, and the color development time was controlled under the microscope; the positive color was brownish yellow, and the color development was terminated by rinsing the section with distilled water. Hematoxylin was re-dyed for 3min and finally sealed by dehydration. Image acquisition was performed using the Pannoramic 250 digital slice scanner (3DHISTECH, Hungary). Aipathwell^®^ (Servicebio^®^) was used to automatically determine positive results based on HSI (Hue, Saturation, Intensity) and classify positive grades: a weak positive light yellow is scored as 1 point. Moderate positive brownish-yellow color, worth 2 points; Strong positive brownish-brown, worth 3 points. Converted the ratio of positive cells and the staining intensity within each section into corresponding values to conduct a comprehensive semi-quantitative analysis of both the depth and quantity of positive tissue immunostaining. H-Score (∑(pi×i) = (percentage of weak intensity cells×1) + (percentage of moderate intensity cells×2) + (percentage of strong intensity cells×3), where i represents the grade classification of positive cells: negative without staining, scored 0 points; Weak positive light yellow, worth 1 point; Moderate positive brownish- yellow color, worth 2 points; A strong positive brownish color worth 3 points. Pi represents the percentage of positive cells of the corresponding grade.

### 2.5 Western blot analysis

The different protein expression levels in the jejunum tissue (50 mg) were detected by WB. The tissues were lysed with RIPA buffer containing 1 mM protease inhibitor and 1 mM phosphatase inhibitor and collected using a cell scraper. Total tissue protein was extracted, and protein concentration was determined using the BCA protein assay kit (Beyotime Biotechnology, Jiangsu, China). An equal amount of cell lysate was separated on a 10% SAL-polyacrylamide gel and transferred to a tailored polyvinylidene fluoride (PVDF) membrane according to the size of the protein. Then the membrane was blocked with Tris-buffered Tween 20 (TBST) with 5% non-fat dry milk at 25°C for 2 h. Subsequently, the membrane was incubated with the following primary antibodies overnight at 4°C: Anti-Actin Rabbit pAb (1:5000), Anti-TBP Rabbit pAb (1:1000), Anti-TLR4 Rabbit pAb (1:1000), Anti-IκBα Rabbit pAb (1:1000), Anti-MYD88 Rabbit pAb (1:1000), and Anti-P-P65 Rabbit pAb (1:1000). Then, the membrane was washed with TBST three times, and incubated the PVDF membrane with goat anti-rabbit secondary antibodies (1:3000) at 25°C for 2 h. Finally, the target protein was detected by an enhanced chemiluminescence system (Boster, China). Densitometric values of protein bands were quantified by Image J software.

### 2.6 Sequencing analysis of 16S rDNA intestinal microbiota structure spectrum

The sequencing analysis of the 16S rDNA intestinal microbiota structure spectrum was based on the Illumina MiSeq sequencing platform. Using the Paired-End sequencing method, small fragment libraries were constructed for sequencing. The optimized sequence (Tags) was obtained by filtering the original sequencing sequence and performing double-ended splicing. Clustered the optimized sequence, divided the OTUs, and obtained their species classification based on the sequence composition of the OTUs. Based on the OTU analysis results, further α-diversity analysis, β-diversity analysis, Lefse analysis of significantly different species, and KEGG function prediction analysis were conducted. The primers used for amplification in this experiment were the 16S rDNA (V3+V4) region primers of bacteria: 341F: 5’-CCTACGGGNGGCWGCAG-3’, 805R: 5’-GACTACHVGGGTATCTAATCC-3’. DNA extraction, PCR amplification, and sequencing were all accomplished in collaboration with Servicebio^®^ Biotechnology Company.

### 2.7 Statistical analysis

All data were presented as Mean ± SEM. Data were analyzed using GraphPad Prism™ software 5.0 (GraphPad Software, Inc., California, USA). One-way analysis of variance (ANOVA) followed by a Dunnett’s post-test was used to determine the difference between the groups. All groups are compared with the model group, * *P* < 0.05, ** *P* < 0.01, *** *P* < 0.001.

## 3 Results

### 3.1 Ellagic acid reduces the viral load in RV−infected Balb/c suckling mice

**Figure 1:**
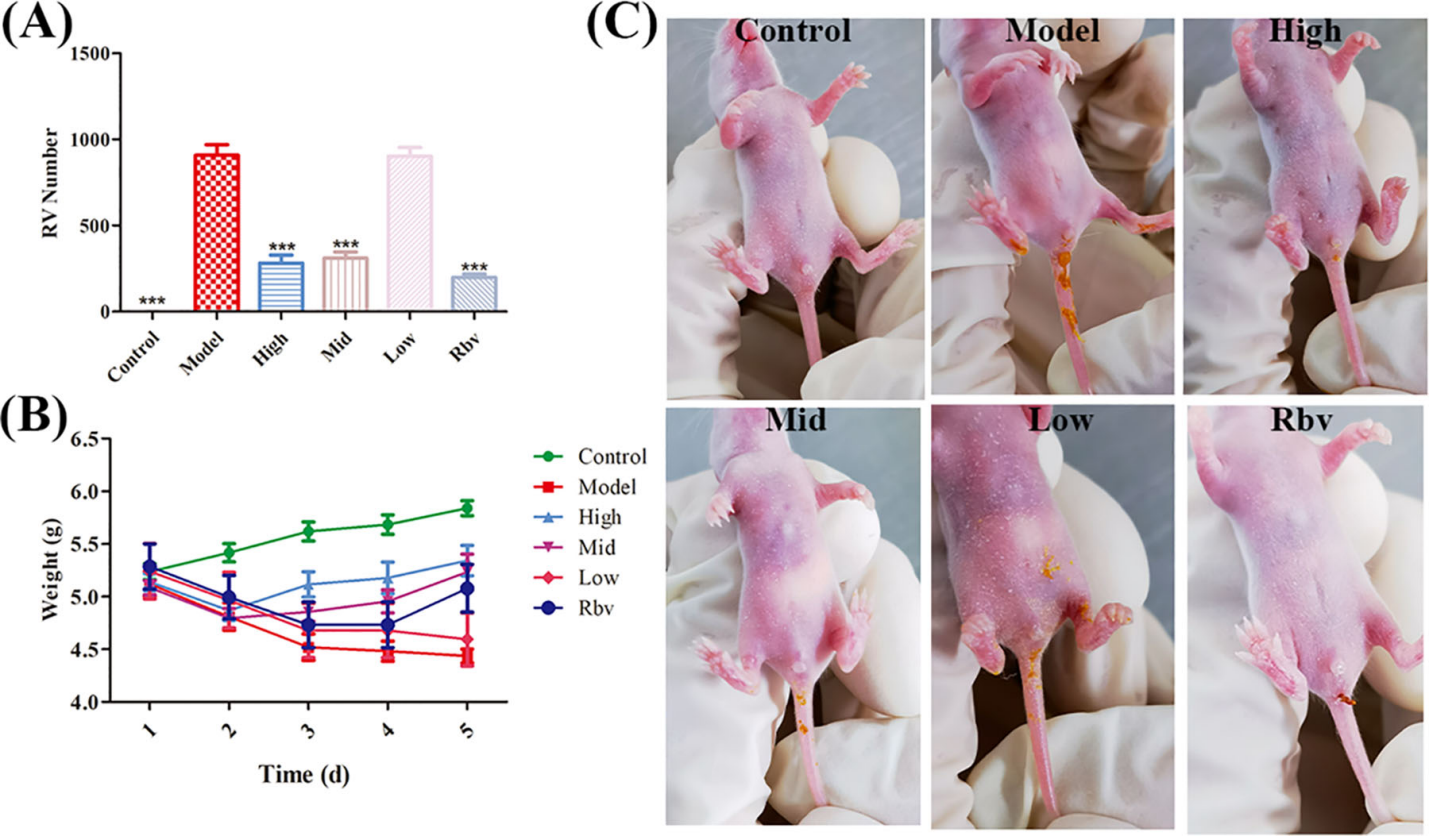
Effects of ellagic acid on RV-infected BALB/c suckling mice after 72 h **(A)** Viral load. **(B)** Body weight. **(C)** Diarrhea in RV-infected Balb/c suckling mice after ellagic acid treatment. *P<0.05, **P<0.01, ***P<0.001.

Three days after treating RV-infected Balb/c suckling mice with ellagic acid and ribavirin, the viral load was analyzed by qPCR to determine the anti-RV efficacy. Compared with the infected group, ellagic acid and ribavirin (**Figure 1A**) significantly (*P* < 0.001) reduced viral load. These results demonstrated that ellagic acid and ribavirin significantly reduced the replication of RV in Balb/c suckling mice. Ellagic acid also exhibited a certain alleviating effect on the weight loss of RV-infected Balb/c mice (**Figure 1B**). The effect of ellagic acid on diarrhea in RV-infected Balb/c suckling mice is shown in **Figure 1C**.

### 3.2 Ellagic acid alleviates intestinal damage caused by RV infection

**Figure 2:**
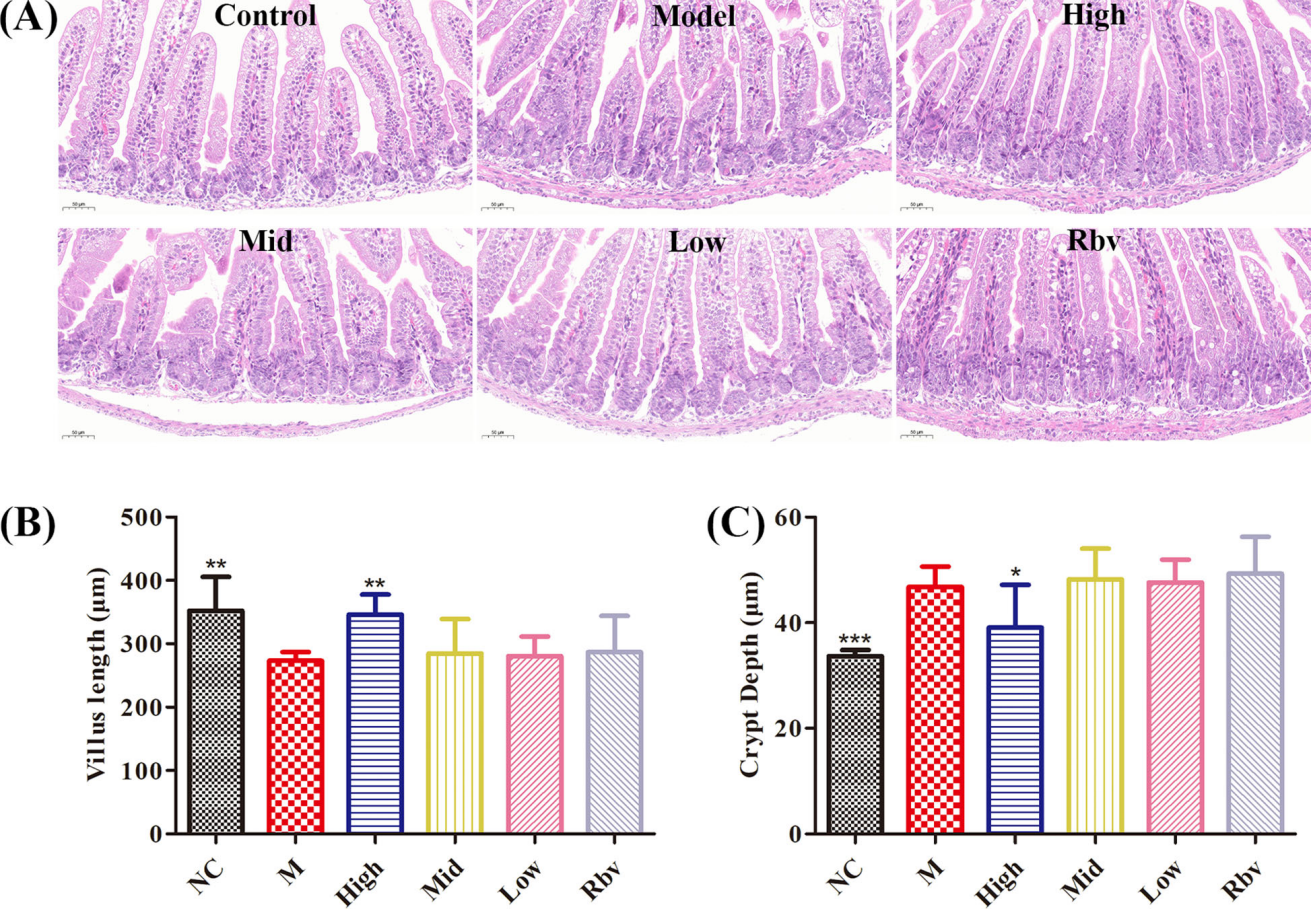
Effects of ellagic acid on intestinal damage **(A)** H&E staining of jejunum tissue after 72h of ellagic acid treatment. **(B)** Villus length. **(C)** Crypt depth. *P<0.05, **P<0.01, ***P<0.001.

**Figure 3:**
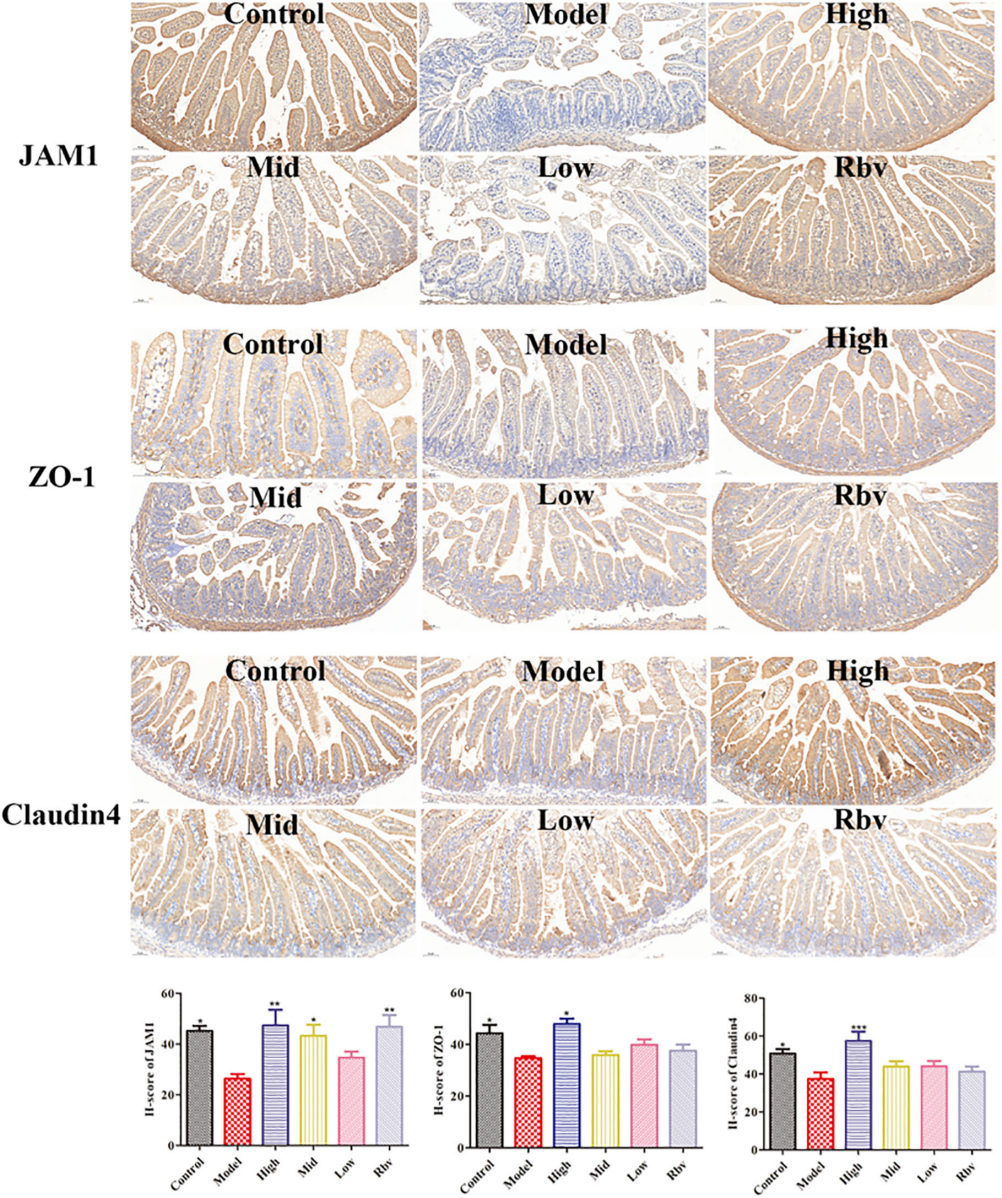
The expression of JAM1, ZO-1 and Claudin4 proteins was detected by IHC, respectively, after ellagic acid treatment.

The histopathological findings showed that compared with the control group, the jejunal tissue in the model group exhibited ruptured villi, shortening, deepened crypts, and distortion, while ellagic acid could alleviate the above symptoms (**Figure 2A**). The villi in the model group were significantly shortened, and the crypts were significantly deepened (*P* < 0.05). After 4 days of ellagic acid treatment, significant (*P* < 0.05) improvements were observed (**Figures 2B, C**). The immunohistochemical results showed that compared with the control group, the expression levels of JAM1, ZO-1, and Claudin-4 in the model group were significantly (*P* < 0.05) decreased and could be reversed after 3 days of ellagic acid treatment (*P* < 0.05, **Figure 3**).

### 3.3 Ellagic acid inhibits the TLR4/NF-κB signaling pathway

**Figure 4:**
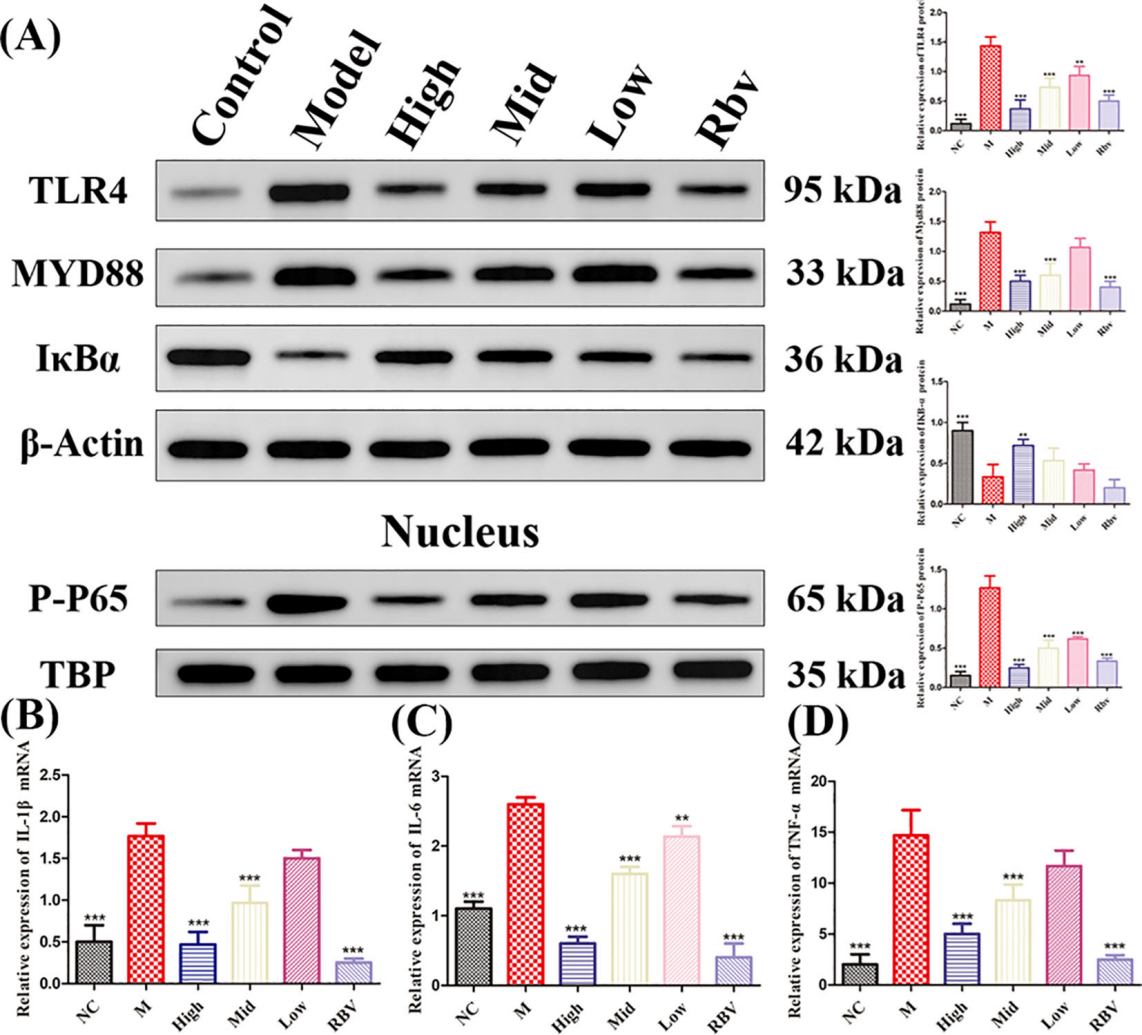
Ellagic acid inhibits the TLR4 signaling pathway to alleviate intestinal damage caused by RV infection. **(A)** The expression of TLR4, MYD88, IκBα, and P-P65 proteins were detected by Western blot. Original blots are presented in **Supplementary Figure 1**. **(B–D)** The expression of *IL-1β*, *IL-6* and *TNF-α* mRNA measured by qPCR. *P<0.05, **P<0.01, ***P<0.001.

The expression of proteins related to the TLR4/NF-κB pathway was detected by WB. Compared with the control group, RV induced the significant (*P* < 0.001) production of TLR4, MYD88, and P-P65 protein, and significantly decreased the expression of IκBα (**Figure 4A**). Compared with the model group, EA significantly decreased (*P* < 0.05) the expression of TLR4, MYD88, and P-P65, while markedly increased (*P* < 0.05) the expression of IκB. The mRNA expression of *IL-1β*, *IL-6*, and *TNF-α* was analyzed by qPCR. Compared with the control group, RV induced the significant (*P* < 0.001) production of *IL-1β*, *IL-6*, and *TNF-α* mRNA (**Figures 4B-D**). Compared with the model group, EA significantly decreased (*P* < 0.001) the expression of *IL-1β*, *IL-6*, and *TNF-α* mRNA.

### 3.4 Alpha diversity analysis and beta diversity analysis

**Figure 5:**
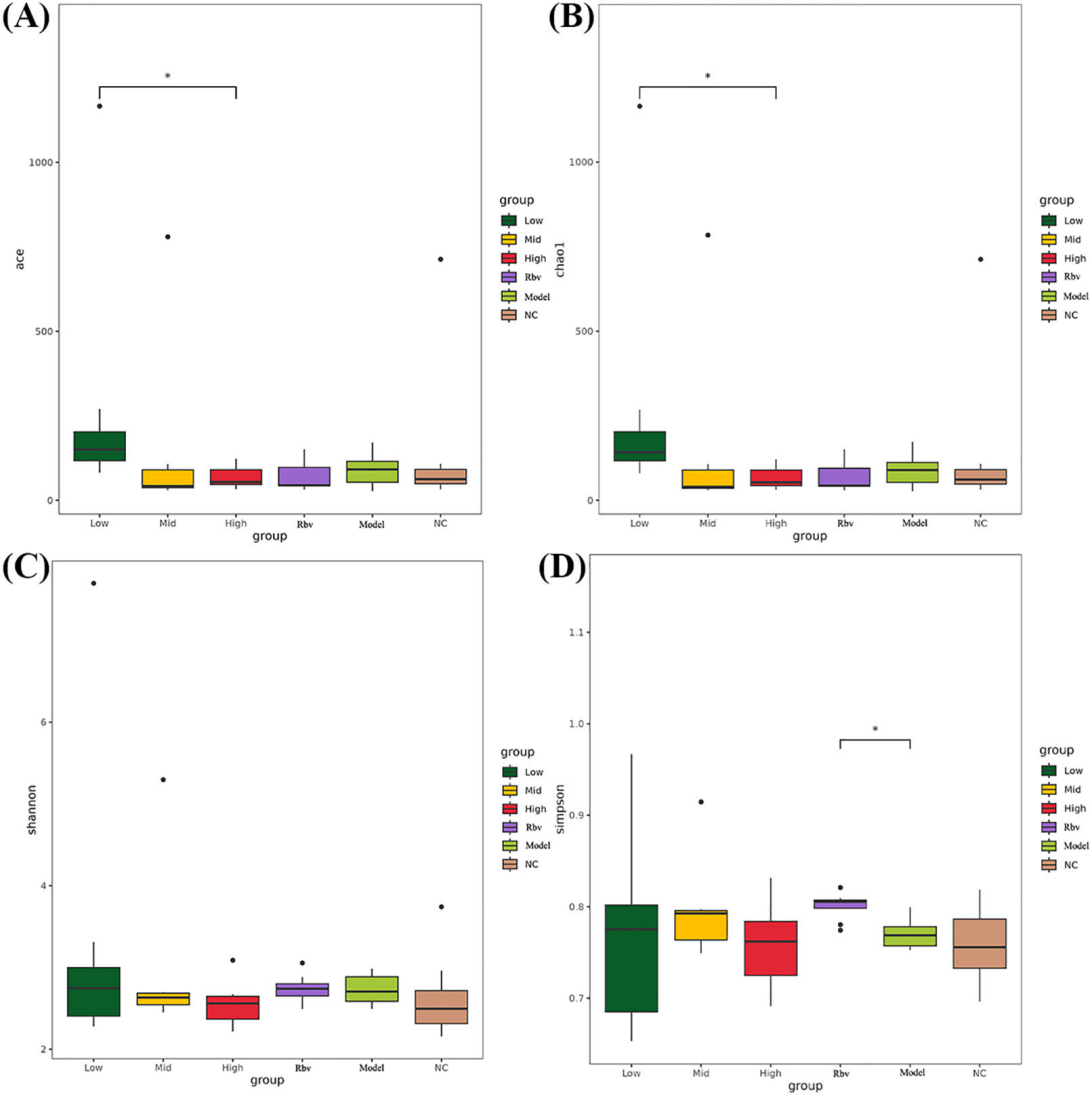
Results of α-diversity analysis. **(A)** Ace index. **(B)** Chao1 index. **(C)** Shannon index. **(D)** Simpson index. *P<0.05, **P<0.01, ***P<0.001.

The results of the α-diversity analysis showed that compared with the control group, there were no significant differences in the ace (**Figure 5A**), chao1 (**Figure 5B**), Shannon (**Figure 5C**), and Simpson (**Figure 5D**) indices in the model group. It indicates that viral infection does not have a significant impact on the overall complexity and diversity structure of the intestinal microbiota in mice. Compared with the high-dose group, ace and chao1 in the low-dose group were significantly increased, indicating that low-dose drugs can significantly enhance the species richness of the intestinal flora in mice.

**Figure 6:**
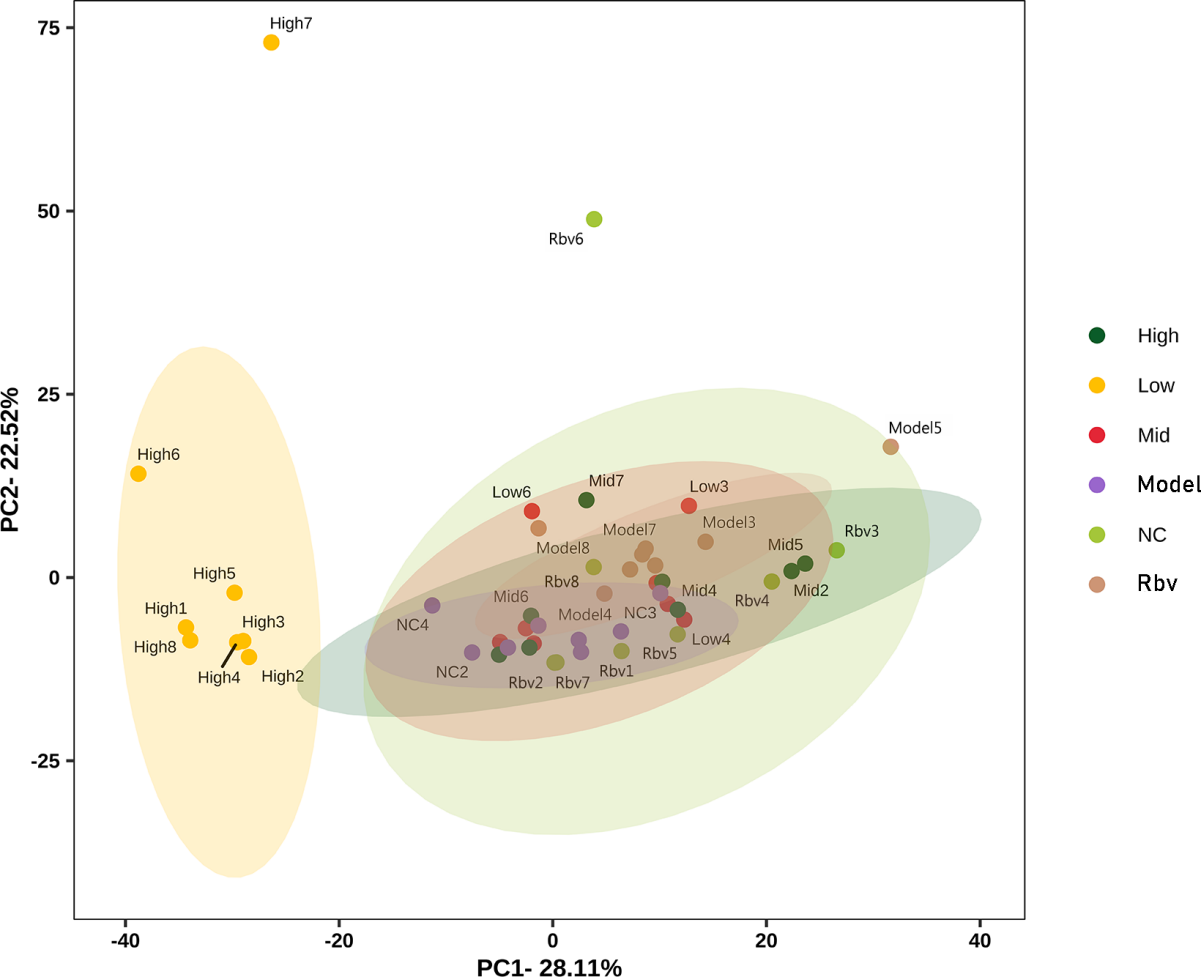
PCoA analysis.

The results of β-diversity analysis showed that samples from the high-dose group clustered independently, while samples from the high-dose group, medium-dose group, low-dose group, model group, and control group clustered together into another cluster (PC1 contribution rate 28.11%, PC2 contribution rate 22.52%, cumulative 50.22%). It indicates that high-dose EA significantly altered the overall community structure of the intestinal microbiota in mice, leading to a systematic shift in the microbial composition between groups (**Figure 6**).

### 3.5 Lefse analysis

**Figure 7:**
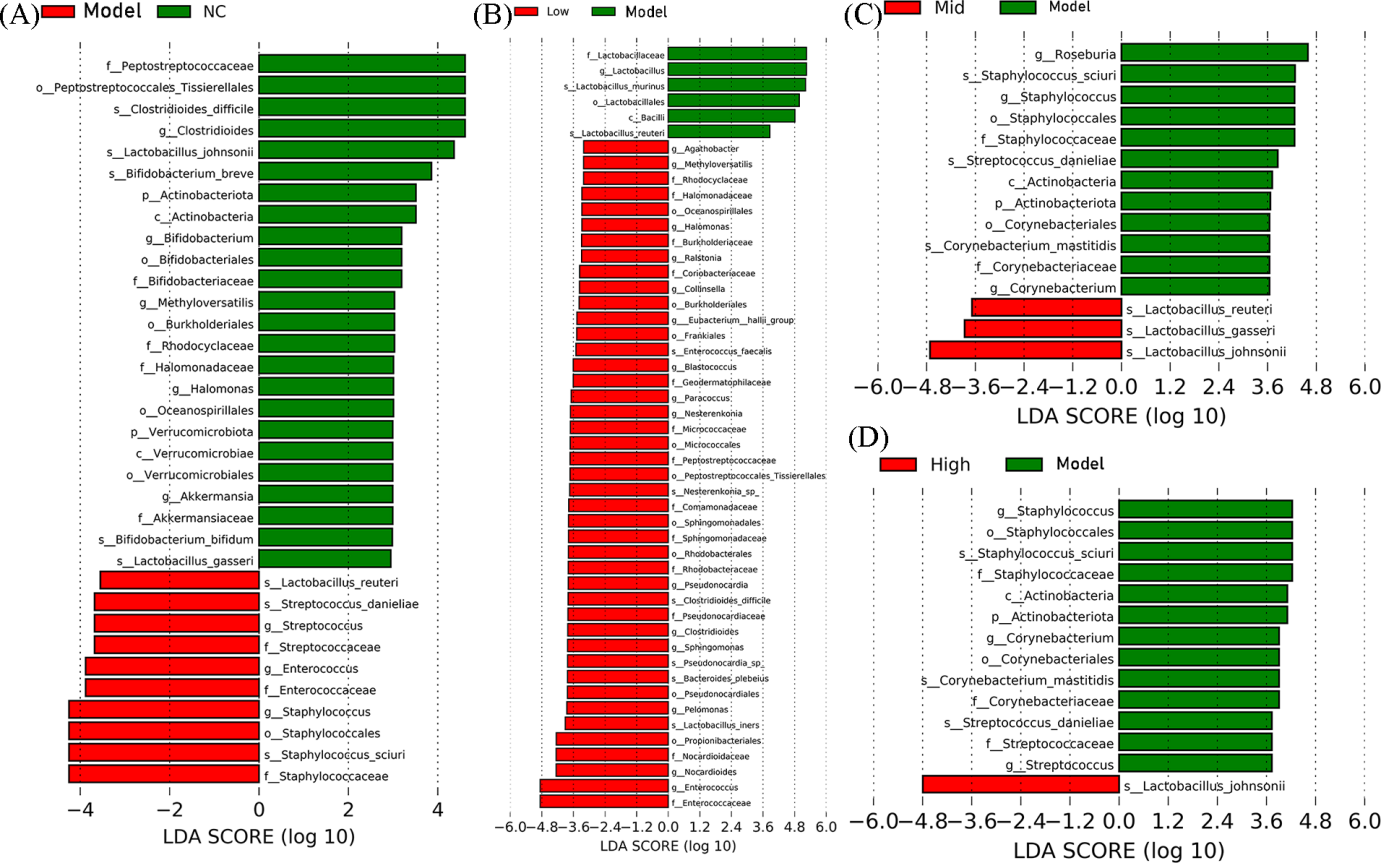
Lefse analysis of significantly differential microbiota. **(A)** Significantly different strains between the NC group and the Model group (P<0.05, LDA>2.0). **(B)** Significantly different strains between the Low group and the Model group (P<0.05, LDA>2.0). **(C)** Significantly different strains between the Mid group and the Model group (P<0.05, LDA>2.0). **(D)** Significantly different strains between the High group and the Model group (P<0.05, LDA>2.0).

**Figure 8:**
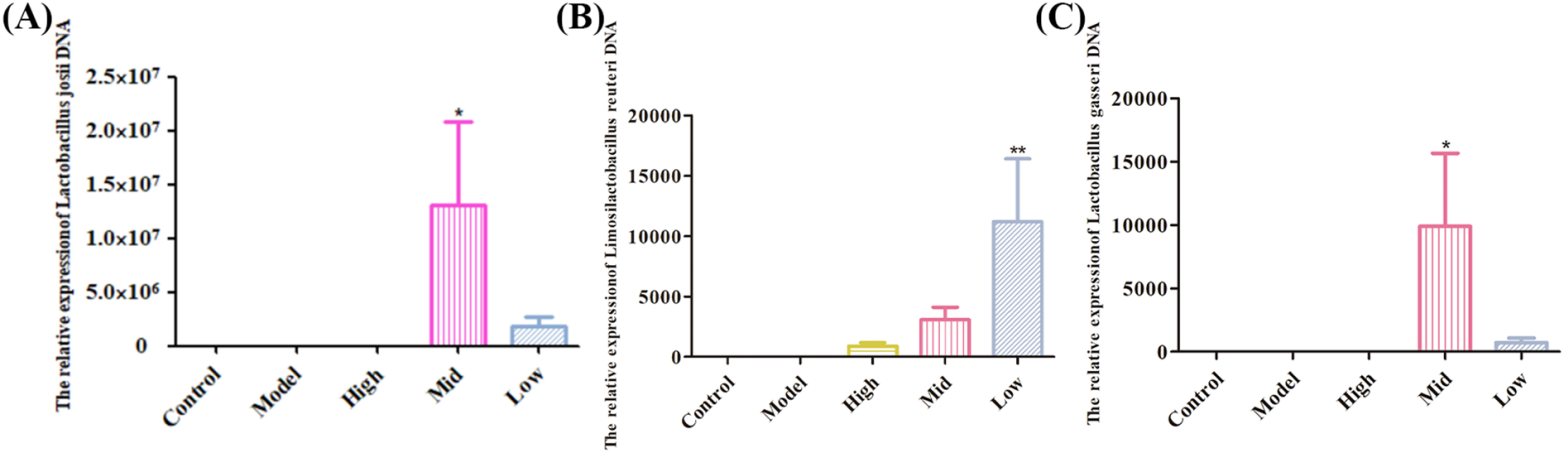
qPCR was used to detect the mRNA of bacteria in the jejunum. **(A)** Lactobacillus johnsonii. **(B)***Lactobacillus reuteri*. **(C)***Lactobacillus gasseri*.

The Lefse analysis results showed that, compared with the control group, virus infection led to a significant (*P* < 0.05) decrease in the abundance of probiotics such as *Lactobacillus-Gasseri*, *Lactobacillus-Johnsonii*, and *Bifidobacterium- Bifidum* in the jejunum of mice (**Figure 7A**). Compared with the model group, the abundance of probiotics such as *Lactobacillus-iners* in the low-dose group was significantly (*P* < 0.05) increased (**Figure 7B**). The abundance of probiotics such as *Lactobacillus-Johnsonii*, *Lactobacillus-Reuteri*, and *Lactobacillus-Gasseri* in the medium-dose group increased significantly (*P* < 0.05) (**Figure 7C**). The abundance of probiotics such as *Lactobacillus-Johnsonii* in the high-dose group increased significantly (*P* < 0.05) (**Figure 7D**). These findings indicate that RV infection can lead to a decrease in the abundance of probiotics such as *Lactobacillus-Johnsonii*. While high, medium, and low doses of the EA can lead to a significant increase in the abundance of Lactobacillus bacteria (*Lactobacillus-Johnsonii*, *Lactobacillus-Reuteri*, and *Lactobacillus-Gasseri*) in the jejunum of mice. It is suggested that EA can exert antiviral effects by regulating probiotics such as *Lactobacillus- johnsonii* in the jejunum of mice. The bacterial DNA of jejunal tissue was detected, and the results demonstrated that a medium dose of EA could significantly (*P* < 0.05) colonize *Actobacillus-johnsonii*, *Lactobacillus-reuteri*, and *Lactobacillus-gasseri* (**Figure 8**).

### 3.6 KEGG analysis

**Figure 9:**
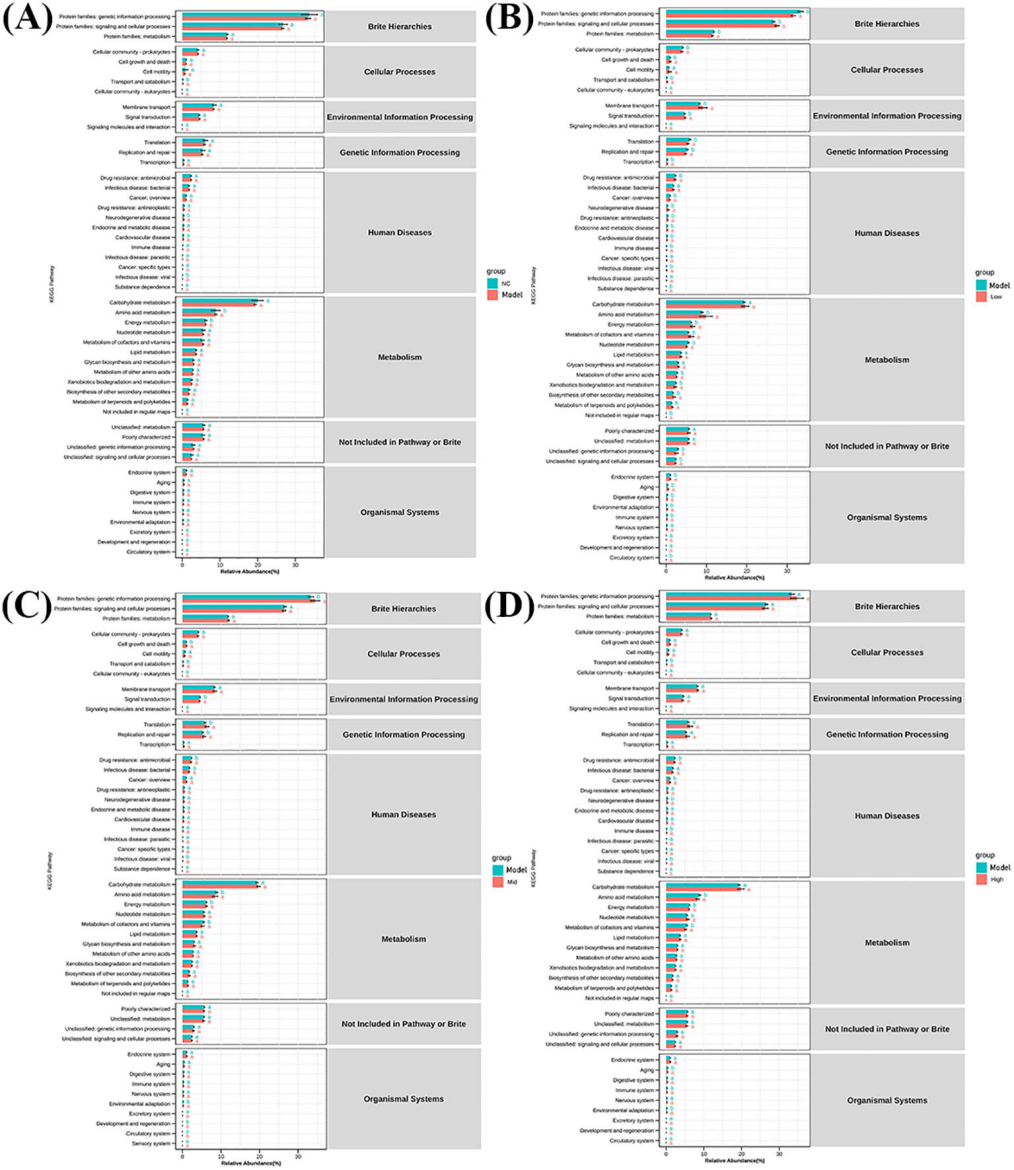
KEGG analysis.

The KEGG analysis results showed that amino acid metabolism and energy metabolism were significantly enriched (*P* < 0.05) between the control group and the model group (**Figure 9A**). Amino acid metabolism, Energy metabolism, Metabolism of cofactors and vitamins pathways were significantly enriched (*P* < 0.05) between the high-dose, medium-dose, and low-dose groups and the model group (**Figures 9B-D**). It indicates that RV infection can lead to a decrease in the abundance of *Lactobacillus-johnsonii* intestinal flora, thereby inducing changes in metabolic pathways such as Amino acid metabolism and Energy metabolism. EA can increase the abundance of intestinal flora such as *Lactobacillus-johnsonii*. It affects the changes of metabolic pathways such as Amino acid metabolism, Energy metabolism, Metabolism of cofactors and vitamins, thereby exerting antiviral effects.

## 4 Discussion

In traditional Chinese medicine, pomegranate peels are often used to treat diarrhea. It contains a specific compound known as ellagic acid, so it has a very high potential value in anti-RV (Xiang et al., 2022). Firstly, we used an RV-infected Balb/c suckling mouse model to verify the potent anti-RV activity of ellagic acid. The results of histopathological sections revealed that ellagic acid could also alleviate intestinal damage caused by RV infection. In the early stage of this study, techniques such as network pharmacology, computational biology, and SPR were utilized to screen out TLR4 as a potential target of ellagic acid against RV, and the two can specifically bind. This was identified as a crucial aspect for investigating the antiviral mechanism (Zheng et al., 2024).

The pathogenic mechanism of rotavirus is to damage intestinal epithelial cells, leading to intestinal villi damage and abnormal proliferation of crypts, which affects the digestion and absorption of carbohydrates and causes osmotic diarrhea (Amimo et al., 2021). The results of H&E showed that ellagic acid could alleviate the shortening of intestinal villi and deepening of crypts caused by RV. Rotavirus-infected cells will release ADP to bind to the P2Y1 receptor on uninfected cells, thereby inducing intercellular calcium waves and activating chloride ion channels to initiate diarrhea (Chang-Graham et al., 2020). After RV infection, the tight junction proteins of IEC significantly decrease. Mature IECs shed in large quantities under the mechanical action of intestinal contents, causing absorption dysfunction and leading to diarrhea (Dong et al., 2023). In addition, the expression of inflammatory factors mediated by TLR4 can also down-regulate the expression of tight junction proteins and disrupt their normal localization (Wardill et al., 2014; Zhao et al., 2025). The results of this study indicate that ellagic acid can significantly alleviate the reduction of tight junction proteins (TJP-1, Claudin-4, and ZO-1) caused by RV infection.

Toll-like receptors (TLRs), as transmembrane inflammatory receptors, are generally involved in mucosal innate immune regulation (McKernan, 2022). TLR4 is a member of the TLR family, capable of specifically recognizing LPS and located in the cell membrane and cytoplasm (Lim and Staudt, 2013). The combined effect of TLR4-dependent Claudin-1 internalization and pro-secret-mediated chloride secretion leads to diarrhea (Wang et al., 2021). In the treatment of gastrointestinal diseases, an increasing number of studies have focused on blocking the TLR4/NF-κB pathway. TLR4 has a significant role in intestinal immunity. After recognizing LPS, TLR4 undergoes oligomerization and recruits its downstream adaptor proteins (such as MyD88, TIRAP, TRIF, TRAM, and SARM) through interaction with the TIR (Toll-interleukin-1 receptor) domain (Lu et al., 2008). Among them, MyD88 plays a significant role downstream of TLR4 signal transduction, and the MyD88-dependent TLR4 pathway is responsible for the transcription of pro-inflammatory cytokines (Laird et al., 2009; Zhe et al., 2022). The results of WB and qPCR showed that ellagic acid could inhibit the TLR4/MyD88/NF-κB signaling pathway in RV-infected Balb/c mice and suppress the expression of inflammatory factors. Therefore, ellagic acid can alleviate intestinal damage caused by RV infection by inhibiting the TLR4/MyD88/NF-κB signaling pathway.

Rotavirus is usually a self-limiting disease with a relatively short course, averaging 7 days. However, secondary intestinal flora disorders can prolong the disease course and aggravate symptoms (Farthing, 2001). The proliferation phenomenon of Gram-negative bacteria after rotavirus infection has been confirmed by multiple studies (Wu et al., 2023). Given the significance of TLR4 in intestinal immunity and mediating LPS recognition, ellagic acid may also alleviate intestinal damage caused by RV infection by inhibiting the response of TLR4 to secondary proliferating Gram-negative bacteria. However, it is worth noting that if TLR4 is targeted, it will inhibit the body’s immune control over Gram-negative bacteria, leading to their abnormal proliferation. Ellagic acid can not only target TLR4 to inhibit the inflammation caused by its response to LPS, but also directly inhibit the proliferation of Gram-negative bacteria (Nunes et al., 2018). Therefore, ellagic acid can alleviate intestinal damage caused by RV infection by reducing the response and proliferation of Gram-negative bacteria.

Surprisingly, ellagic acid has no inhibitory effect on probiotics and bifidobacteria, especially *Lactobacillus rhamnosus GG* and *Bifidobacterium infantis* (Lee et al., 2014). Probiotics are currently widely used to improve adverse reactions to rotavirus vaccination and intestinal damage caused by RV infection (Parreno et al., 2022; Michael et al., 2022; Vlasova et al., 2016). *Lactobacillus reuteri* shows good safety and reliability in the treatment of rotavirus gastroenteritis in children (Markovinović et al., 2020). The results of this study showed that RV infection can lead to a decrease in the abundance of probiotics such as *Lactobacillus johnsonii*. Ellagic acid can lead to a significant increase in the abundance of Lactobacillus bacteria (*Lactobacillus johnsonii*, *Lactobacillus reuteri*, and *Lactobacillus gasseri*) in the intestinal tract of mice. Based on the dual nature of ellagic acid’s role in the complex intestinal microecology, it is suggested that ellagic acid can exert an anti-RV effect by regulating probiotics such as *Lactobacillus johnsonii* in the mouse intestine. *Lactobacillus reuteri* not only has a relieving effect on diarrhea caused by rotavirus, but also can alleviate intestinal damage (Wu et al., 2020; Mao et al., 2023). The administration of *Lactobacillus gasseri* by the mother can stimulate the secretion of IgA in breast milk to prevent diarrhea caused by rotavirus (Kadooka et al., 2012). The interaction relationship between *Lactobacillus johnsonii* and rotavirus is currently unclear, but it can alleviate enteritis by inhibiting the TLR4/NF-κB signaling pathway, which also indicates its potential for anti-RV and alleviating intestinal damage (Chen et al., 2021).

## 5 Conclusion

Ellagic acid improves intestinal injury induced by RV infection through an “immune-microbiota” bidirectional regulatory effect. It significantly reduces the overexpression of inflammatory factors caused by RV infection by inhibiting the TLR4/NF-κB signaling pathway, thereby alleviating the intestinal inflammatory response.

## Data Availability

The datasets presented in this study can be found in online repositories. The names of the repository/repositories and accession number(s) can be found in the article/**Supplementary Material**.
