# Supplementary figures and images for: Ellagic acid mitigates rotavirus-induced intestinal injury via bidirectional “immune-microbiota” regulatory effect
Source: Front Cell Infect Microbiol. 2025 Dec 29;15:1686918. doi: 10.3389/fcimb.2025.1686918 (PMC12791041; doi:10.3389/fcimb.2025.1686918)

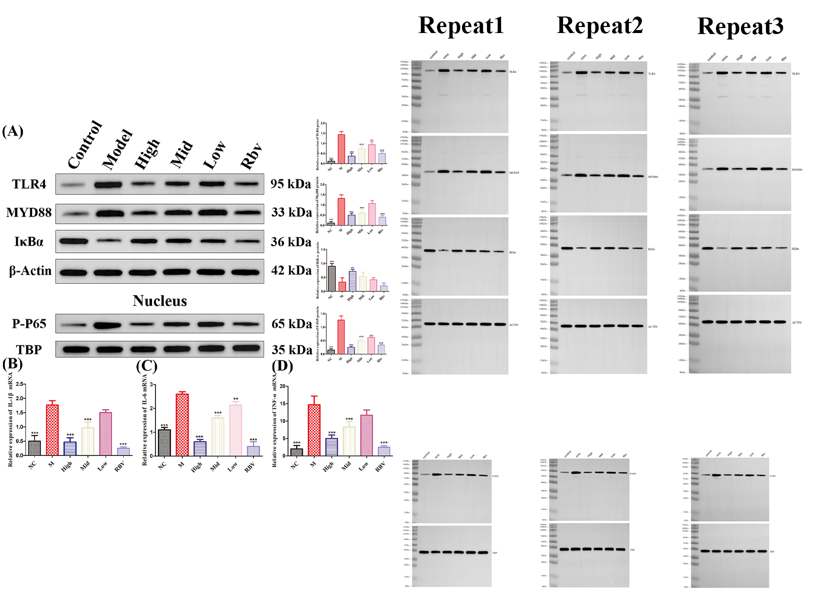

Supplement: Supplementary file 1 [file Image1.tif]
